# Comparative Copper Resistance Strategies of *Rhodonia placenta* and *Phanerochaete chrysosporium* in a Copper/Azole-Treated Wood Microcosm

**DOI:** 10.3390/jof8070706

**Published:** 2022-07-04

**Authors:** Gaurav Pandharikar, Kévin Claudien, Christophe Rose, David Billet, Benoit Pollier, Aurélie Deveau, Arnaud Besserer, Mélanie Morel-Rouhier

**Affiliations:** 1 Université de Lorraine, INRAE, IAM, 54000 Nancy, France; gaurav-girish.pandharikar@inrae.fr (G.P.); kevin.claudien@univ-lorraine.fr (K.C.); 2 Université de Lorraine, AgroParisTech, INRAE, Silva, 54000 Nancy, France; christophe.rose@inrae.fr; 3 Université de Lorraine, CNRS, LIEC, 54000 Nancy, France; david.billet@univ-lorraine.fr; 4 Pôle de Compétences Chimie Analytique Environnementale, ANATELo, Université de Lorraine, 54000 Nancy, France; 5 Unit INRAE, UR 1138, BEF, 54280 Champenoux, France; benoit.pollier@inrae.fr; 6 Université de Lorraine, INRAE, LERMAB, F-54000 Nancy, France; arnaud.besserer@univ-lorraine.fr

**Keywords:** copper, wood, detoxification, ligninolytic fungi, oxalate, biosorption

## Abstract

Copper-based formulations of wood preservatives are widely used in industry to protect wood materials from degradation caused by fungi. Wood treated with preservatives generate toxic waste that currently cannot be properly recycled. Despite copper being very efficient as an antifungal agent against most fungi, some species are able to cope with these high metal concentrations. This is the case for the brown-rot fungus *Rhodonia placenta* and the white-rot fungus *Phanerochaete chrysosporium*, which are able to grow efficiently in pine wood treated with Tanalith E3474. Here, we aimed to test the abilities of the two fungi to cope with copper in this toxic environment and to decontaminate Tanalith E-treated wood. A microcosm allowing the growth of the fungi on industrially treated pine wood was designed, and the distribution of copper between mycelium and wood was analysed within the embedded hyphae and wood particles using coupled X-ray fluorescence spectroscopy and Scanning Electron Microscopy (SEM)/Electron Dispersive Spectroscopy (EDS). The results demonstrate the copper biosorption capacities of *P. chrysosporium* and the production of copper-oxalate crystals by *R. placenta*. These data coupled to genomic analysis suggest the involvement of additional mechanisms for copper tolerance in these rot fungi that are likely related to copper transport (import, export, or vacuolar sequestration).

## 1. Introduction

Wood is an organic composite that is mainly characterized by its hygroscopic behaviour, orthotropic composition, and variable natural durability [1]. Wood can be considered as an ecological construction material because it is renewable, sustainable, and provides a solution to CO_2_ sequestration [2]. However, the susceptibility of timber to both biotic (fungi, bacteria, insects) and abiotic degradation (UV, erosion) when used in outdoor terrestrial environments may be a limitation [3]. Direct soil contact is one of the most severe exposure situations that wood can be subjected to. Indeed, in-ground wood is permanently wet and stays in direct contact with wood-degrading organisms such as fungi and bacteria, which, in turn, become well established and can proliferate through the readily available nutrient sources in the wood [4]. To protect timber against microbes at the industrial scale, wood is commonly impregnated with copper (Cu)-based preservatives [2,5]. The most widely used are chromium copper salts, which protect timber for 20 years if properly impregnated by vacuum pressure treatments.

Wood preservative formulations that are currently found on the market include ACQ (alkaline Cu quaternary), CA (Cu-azole), Cu-citrate, and Cu-ethanolamine [6,7]. Among these, Tanalith E^®^, a Cu-Azole-based compound, is the most widely used wood preservative in Europe [8]. The antifungal activities of CA compounds rely on the complementary effects of azoles that inhibit the fungal ergosterol biosynthesis pathway [6,9,10] and the toxicity of high doses of Cu. The main effects of an excess of free cytosolic Cu ions are the inactivation of metalloenzymes by metal displacement, the perturbation of Fe-S cluster assembly, and the generation of reactive oxygen species (ROS) through Fenton chemistry, causing biological damage to the fungal cell [11,12].

However, some wood-decay fungi exhibit high detoxification and tolerance properties against Cu-based preservatives and are able to cope with high amounts of Cu [13,14]. This could lead to resistance to Cu-based antifungal compounds, thus making them inefficient to protect wood against fungal decay. On the other hand, these abilities to resist high Cu concentrations may be exploited for the detoxification of treated wood waste. Indeed, while being essential for wood preservation, these harmful wood preservatives pose a significant threat to the ecosystem and human health when treated wood becomes waste. Currently, it is not possible to recycle this biomass due to the toxicity of the compounds used for its preservation. Nowadays, wood biomass is treated by specific incineration, pyrolysis, and gasification techniques. Although these methods of chemical disposal are low-cost and efficient, they still release toxic pollutants and other harmful components into the atmosphere that potentially damage the environment and public health [15]. To overcome this problem, alternative sustainable solutions to treat wood waste need to be developed. In recent years, some studies have shown that fungi may be used as biocatalysts for copper decontamination in wood waste [16]. These fungi rely on their ability to both leach Cu from the wood and to resist its toxicity.

The cellular and molecular mechanisms responsible for Cu tolerance have been described in ascomycetes. Two major mechanisms have been proposed for Cu detoxification. One of them, which has been well described in *Saccharomyces cerevisiae,* relies on Cu sequestration by metallothioneins [17,18]. The second mechanism relies on Cu extrusion by Cu-transporting ATPases [19]. Few data are available in fungi, but in human, Cu-transporting ATPases change their location from the trans-Golgi compartment to the cell membrane in response to Cu toxicity to act as export pumps conferring copper resistance [20]. Other mechanisms such as the down regulation of Cu importers, ion sequestration into the vacuole, the production of extracellular chelators, or cell wall biosorption could be involved [11].

In contrast to ascomycetes, the molecular mechanisms of Cu homeostasis and resistance mechanisms to preservatives are unexplored in Basidiomycetes. As some of them can cope with high amounts of Cu through the detoxification of copper via the production of copper oxalate, bioleaching, and/or biosorption activities [21,22], they may thus be exploited for the detoxification process of treated wood waste. In the present work, we aimed to decipher the strategies used by brown-rot *Rhodonia placenta* and white-rot *Phanerochaete chrysosporium* fungi, which are known to have high resistance to Cu, to colonize pine wood treated with Tanalith E. We combined experimental and in silico approaches to (i.) identify how the two fungi cope with toxic levels of Cu in the presence of azoles and (ii.) identify the potential molecular players involved in Cu homeostasis and resistance. For this purpose, we have developed a fungal microcosm to work directly on treated wood waste as it is released after industrial use. We used it to test the growth of the two fungi on industrial wood-treated sawdust. We then measured the levels of Cu in the wood, fungal hyphae, and liquid media by coupling X-ray fluorescence spectroscopy (XRF), Inductively Coupled Plasma Atomic Emission Spectrometry (ICP-OES), and Scanning Electron Microscopy (SEM)/Electron Dispersive Spectroscopy (EDS) to determine the fungal ability to detoxify treated wood. In parallel, comparative genomics were used to explore the potential mechanisms used by the two fungi to cope with Cu toxicity at the cellular level. Our results indicate that the two fungi use different strategies to cope with Tanalith E toxicity.

## 2. Material and Methods

### 2.1. Microcosm Set-Up

A biological microcosm was developed to grow *Rhodonia placenta* (Fr.) Niemelä, K.H. Larss. & Schigel (*Rp*) (older names: *Oligoporus placenta*, *Postia placenta*) and *Phanerochaete chrysosporium* RP78 (Burds.) Hjortstam & Ryvarden (*Pc*) in submerged cultures with treated wood. The wood samples were collected from pine wood planks that were industrially impregnated (autoclave class IV impregnation) with Tanalith E3474 preservative product, raising the concentration within the wood to 16.7 kg/m^3^. Tanalith E3474 (Arch Timber Protection Ltd., Castleford, UK) is a commercial formulation composed of 16.4% *w*/*w* copper (copper (II) carbonate–copper (II) hydroxide 1:1), 0.18 % *w*/*w* tebuconazole, and 0.18% *w*/*w* propiconazole. After treatment, the wood was dried, and samples were milled with a cutting mill SM 100 (Retch) to obtain particles with a size between 0.5 and 2 mm. To remove residual unbound products within the sawdust, three mechanical leaching steps were performed over 24 h in ultrapure water by continuous magnetic stirring in 1 L flasks. Subsequently, sawdust was dried overnight at 80 °C in an oven. Parallelly fungal preculture was prepared in 1% malt broth by adding one 7 mm fungal plug per flask and kept at 28 °C in an incubator for 4 days. Biological microcosm prepared in Erlenmeyer flasks containing 25 mL of 1 % malt medium and 2% sawdust (treated sawdust (TSD) or non-treated sawdust (NTSD)) were autoclaved (120 °C—20 min).The fungal precultures that had been grown for 4 days were inoculated in the autoclaved microcosm containing sawdust. Subsequently, the biological microcosm was incubated at 28 °C for ten days and with shaking at 80 rpm shaking for *P. chrysosporium* and without shaking for *R. placenta*.

### 2.2. Respiration Tests

To evaluate the biological activity of *R. placenta* and *P. chrysosporium* in the presence of NTSD and TSD, a fungal respiration assay was conducted. Sealed flasks containing 1% malt medium and 2% leached sawdust were inoculated with four days grown fungal precultures. Fungal activity was followed using a carbometer (LAMBDA laboratory instruments), allowing for the non-destructive quantification of the CO_2_ released during a ten-day growth period. A 120 mL amount of air was harvested every two days and replaced with new 0.2 µm filtered atmospheric air to avoid anoxia in the system. Five biological replicates were used.

### 2.3. X-ray Fluorescence Spectroscopy and Inductively Coupled Plasma Atomic Emission Spectrometry for Copper Quantification in the Liquid Phase

To quantify the precise amount of Cu leached by the fungi in the liquid phase of the microbial microcosms, X-ray Fluorescence Spectrometry (XRF) and Inductively Coupled Plasma Atomic Emission Spectrometry (ICP-OES) were coupled. At 10 days post inoculation, the liquid phase was separated from the sawdust colonized by the fungus by centrifugation at 8350× *g* for 15 min. For the quantification of Cu by XRF (Thermo Scientific, Waltham, MA, USA), 500 µL of the liquid phase was diluted with 1% malt in 2 mL polypropylene cups that were transparent to X-rays. Spectra were analysed using the UniQuant (Thermo Scientific) program after calibration according to the manufacturer’s instructions using different elements. For Cu quantification by ICP-OES (Agilent 720/725 ICP-OES), the liquid phase was filtered through 0.45 μm polytetrafluoroethylene filters, and 10 mL samples were directly used for Cu quantification. A standard curve was prepared using commercially available Cu standard solutions (Merck). Prior to analysing the main experimental samples, semiquantitative analyses were performed to determine the accurate quantity of Cu at highest and lowest precision levels in the biological samples. The quantification results were generated through ICP Expert II (Agilent) software and were further analysed. Three and six biological repeats were performed for the XRF and ICP analyses, respectively.

### 2.4. Scanning Electron Microscopy (SEM) and Electron Dispersive Spectroscopy (EDS) Microanalyses for Copper Quantification in Sawdust and Fungal Hyphae

Coupling electron microscopy with microanalysis (SEM-EDS) allowed for the amount of Cu inside the wood and inside the fungal hyphae to be determined independently. Following 10 days of incubation in the microcosm, the sawdust colonized by the fungus was separated from the liquid phase by centrifugation at 8350× *g* for 15 min. Wood chips corresponding to the solid sawdust/fungus samples were slowly desiccated overnight in a freeze dryer (FreeZone6literbenchtop, Labconco, Kansas City, MO, USA). Subsequently, wood chips were press-stuck on stubs using a conductive carbon cement (LEIT_C, agar scientific) and dried at 50 °C for one hour in oven. A canned air duster was used to ensure the steadiness of the samples on the stubs. Samples were coated with two layers of carbon (10 nm final carbon layer thickness) using four high-current pulses on carbon threads (ACE 600, Leica microsystems, Wetzlar, Germany). Samples were initially observed using a Field Emission Gun SEM (FEGSEM-SIGMA HD-VP; Zeiss, Oberkochen, Germany) placed in high-vacuum mode (10^−4^ Pa), at a 20 kV high-acceleration voltage, a 1 nA current beam, and a 9 mm working distance (requisite analytical distance) in the way to identify and select Sites of Interest (SI). The numeric Back Scattered Detector signal (QBSD; Zeiss) was used to acquire all SI images, and further EDS analyses were performed with a spectrometer (EDS-SDD 80 mm² detector; Oxford instruments, Abingdon, UK) for each SI (three to nine SI per sample; ten microanalyses per SI). Each obtained spectrum was deconvoluted by the software algorithm (INCA software, Oxford Instruments, Abingdon, UK), and the results produce the estimation of the semi-quantitative mass fraction of Cu in the sample. To distinguish between the copper and calcium inside different-coloured crystals, INCA mapping software was used. The reliability of the data relies on the repeatability of the measurements: up to 120 spectra were obtained for each sample condition, and 3 biological samples were prepared for each condition (Refer to Supplemental Data S1 for more details on sampling).

### 2.5. High Performance Liquid Chromatography for Soluble Oxalate Quantification

For the quantification of soluble oxalate, samples were harvested ten days post inoculation and centrifuged at 8346× *g* for 15 min. The liquid phase of the samples was harvested and filtered with 0.2 μm PTFE (polytetrafluoroethylene) filters. Filtered samples were processed via high-pressure ion chromatography (HPIC) ICS-5000+ (thermo) coupled with ab ISQ EM single-quadrupole mass spectrometer (MS). Oxalate was retained on the Thermo IonPac AS11-HC 2 × 250 mm column set and was detected with a conductimetric detector. The separation was enhanced with mass spectrometric detection in SIM mode. The hydroxide eluent started with a low concentration (1 mM KOH) to separate the weakly retained anions. After maintaining this concentration for 8 min, the eluent concentration was gradually increased to elute the retained anions more strongly. The KOH concentration was increased to 30 mM at 28 min, during which time the oxalate eluted (retention time 22 min). The oxalate’s limit of quantification was 50 µg/L. A 10 millilitre amount of 0.2 µm filtered liquid phase was used for quantification. Three biological replicates from each treatment were performed.

### 2.6. Statistical Analyses

All statistical analyses performed with R studio and GraphPad Prism version 7.04. All figures were generated through GraphPad Prism 7.04. All experimental data were expressed as mean ± s.e. To test the normality of the samples, the Shapiro–Wilk (W) test was performed. For the fungal respiration assay, two-way ANOVA was performed. Differences between the conditions over the time period were tested using Tukey’s post hoc multiple comparison test. Data generated on the ICP, XRF, and SEM-EDS were analysed using one-way ANOVA. Then, Tukey’s post hoc multiple comparison test was performed to identify possible statistical differences between the different conditions.

### 2.7. Genome Mining to Compare Copper Related Genes in R. placenta and P. chrysosporium

Gene sequences related to Cu homeostasis were first searched for within the *Saccharomyces* genome database (Available online: https://www.yeastgenome.org/ (accessed on 9 February 2022) using “copper” as a keyword. The list was then manually curated to the keep genes coding for proteins involved in Cu transport, chelation, and reduction and the regulation of gene expression. The amino acid sequences of these candidates were used as a template for a Blast search using *R. placenta* MAD-698-R-SB12 v1.0 and the *P. chrysosporium* RP78 v2.2 genomes from the Mycocosm of the Joint Genome Institute database (Available online: https://mycocosm.jgi.doe.gov/mycocosm/home (accessed on 9 February 2022)). To complete the analysis, a search using “copper” as keyword was also performed on both genomes. The obtained sequences were compared to the ones retrieved using the Blast search tool. Additional sequences were manually checked for annotation and were included in the dataset. Finally, each sequence was blast back to the corresponding genome to retrieve all isoforms for a specific gene family. Evolutionary analyses were conducted in MEGA X [23]. The evolutionary history was inferred using the neighbor-joining method [24]. The trees were drawn to scale, with branch lengths in the same units as those of the evolutionary distances used to infer the phylogenetic tree. The evolutionary distances were computed using the p-distance method [25] and are in the units of the number of amino acid differences per site. All ambiguous positions were removed for each sequence pair (pairwise deletion option).

## 3. Results

### 3.1. Efficient Growth of Both the Brown-Rot Rhodonia Placenta and the White-Rot Phanerochaete Chrysosporium on Tanalith E3474-Treated Wood

**Figure 1:**
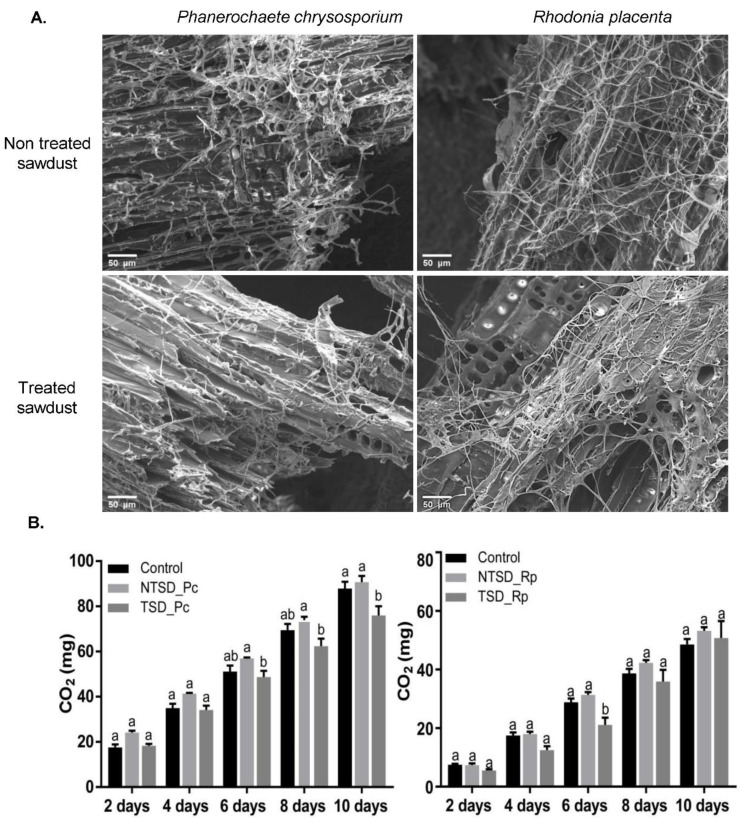
Fungal colonization of non-treated and treated sawdust: (**A**) Scanning Electron Microscopy images of *P. chrysosporium* and *R. placenta* colonization of treated (TSD) and non-treated (NTSD) sawdust after 10 days; (**B**) cumulative CO_2_ production for *P. chrysosporium* and *R. placenta* grown on malt (control), non-treated (NTSD_Pc/NTSD_Rp), and treated (TSD_Pc/TSD_Rp) sawdust over 10 days; mean ± s.e., *n* = 5; two-way ANOVA and Tukey’s post hoc test (*p*-value ≤ 0.05). Identical letters indicate no significant differences in the respiration between treatments at a given time point.

*Phanerochaete chrysosporium* and *R. placenta* were cultivated in the presence of non-treated or treated pine sawdust supplemented with a small volume of malt medium. The supplementation with malt aimed to provide the fungi with a carbon source and other nutrients to remove the efficiency of the wood-degradative systems and to only focus on the detoxification of the treated wood. The system was set-up to allow the whole analysis of both the liquid phase and the solid phase composed of a complex embedded structure of hyphae and wood particles (Figure S1). Scanning Electron Microscopy images of the solid phase clearly showed that both fungi were able to colonize treated wood particles, without obvious modifications to the hyphae morphology compared to the non-treated sawdust condition (Figure 1A). To assess the metabolic activity of the fungi on the treated sawdust, a respiration assay was performed from day 2 to 10 after fungal inoculation on sawdust (Figure 1B). The cumulative amount of CO_2_ released at day 10 was lower in TSD compared to the control without sawdust and NTSD samples for *P. chrysosporium*. The overall amount of CO_2_ released per day was relatively constant all along the kinetics (around 10 mg for *R. placenta* and 17 mg for *P. chrysosporium*). Globally, the presence of Tanalith E within the wood substrates had little to no impact on the primary metabolism of *R. placenta* and *P. chrysosporium* or on their ability to colonize wood particles.

### 3.2. Comparative Copper Bioleaching Ability of R. placenta and P. chrysosporium from Tanalith E3474 Treated Wood

**Figure 2:**
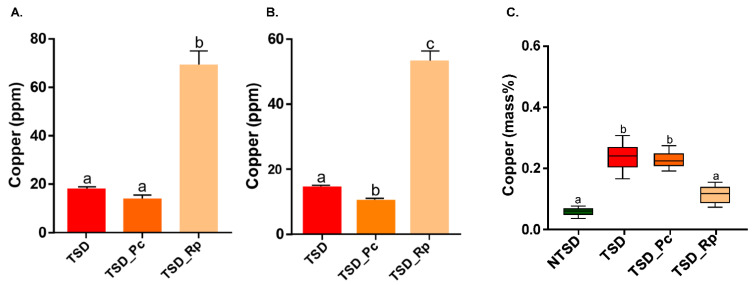
Copper quantification in liquid and solid phases of the microcosm after 10 days of *P. chrysosporium* and *R. placenta* growth with TSD (TSD_Pc/TSD_Rp). Amount of copper retrieved in the liquid phase measured by XRF (**A**) and ICP-OES (**B**). TSD represents the control without fungus. XRF = mean ± s.e., *n* = 3; ICP-OES = mean ± s.e., *n* = 6. (**C**) Copper quantified in wood by SEM-EDS microanalyses. The amount of copper was expressed as the estimated mass fraction of copper in the sample (mean ± s.e., *n* = 9; one-way ANOVA and Tukey’s post hoc test (*p*-value ≤ 0.05), different letters to show statistically significant differences).

First, total Cu was quantified in NTSD and TSD by X-Ray fluorescence before and after leaching. A total of 2.78 g of copper/kg of dry wood (2780 ppm) was measured in the starting TSD material. The water leaching steps released 0.7 % of the Cu measured in TSD. In NTSD, almost no Cu was detected. Cu levels in the liquid phase of the microcosm were quantified at 10 days of culture by both XRF and ICP-OES (Figure 2A,B). As expected, negligible amounts of Cu were detected in the liquid phase of the non-treated sawdust setup (<2 ppm, data not shown). For TSD, a control without fungi was analysed to estimate the amount of Cu leached by the malt medium and the sterilization step. Less than 20 ppm was released in the culture medium without fungi (Figure 2A,B).

**Figure 3:**
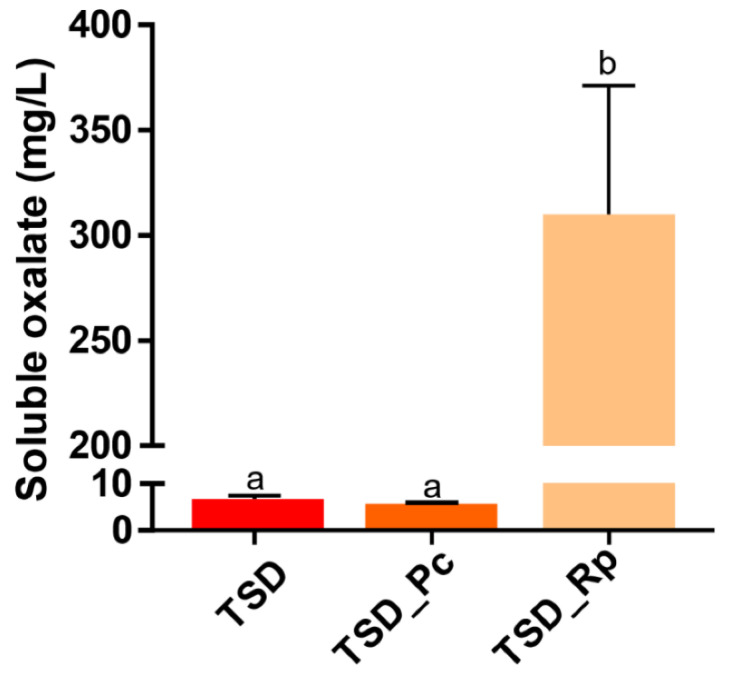
Soluble oxalate quantification in the liquid phase of the microcosm after 10-day culture of *P. chrysosporium* and *R. placenta* with TSD (TSD_Pc/TSD_Rp). TSD represents the control without fungus (mean ± s.e., *n* = 3, one-way ANOVA and Tukey’s post hoc test (*p*-value ≤ 0.05), different letters to show statistically significant differences).

In the presence of *P. chrysosporium* (TSD_Pc), a decrease of about 25% in the amount of Cu in the liquid phase was measured compared to the TSD condition without fungi. This difference was observed using both methods; however, it was only statistically significant for the ICP-OES analysis. Additionally, Cu was quantified in the sawdust by Scanning Electron Microscopy with Energy Dispersive Spectroscopy (SEM-EDS). The Cu levels in the TSD_Pc samples were similar to the control without mycelium (TSD) (Figure 2C). Taken together, these results suggest that *P. chrysosporium* has little ability to bioleach Cu from treated sawdust in liquid medium. In contrast, a strong release (about 38 to 51 ppm) of Cu was measured in the liquid phase for *R. placenta* (Figure 2A,B), which was correlated with a significant decrease (about 54 %) in Cu in the treated sawdust particles (TSD_Rp) (Figure 2C). The Cu level in TSD_Rp was relatively similar to that of NTSD, showing successful Cu leaching. It is well-documented that metal bioleaching in *R. placenta* is mainly ensured by organic acid secretion by fungi [26,27]. Thus, soluble oxalate was quantified in the liquid phase of the culture system at 10 days (Figure 3). The amount of soluble oxalate reached 300 mg/L for *R. placenta* after 10 days of culture in treated sawdust, suggesting that the high Cu leaching efficiency of *R. placenta* relied on its high oxalate production. In contrast, low levels (6 mg/L) of soluble oxalate were secreted by *P. chrysosporium*.

### 3.3. SEM-EDS Based Comparative Analysis of Copper Detoxification Strategies between R. placenta and P. chrysosporium

**Figure 4:**
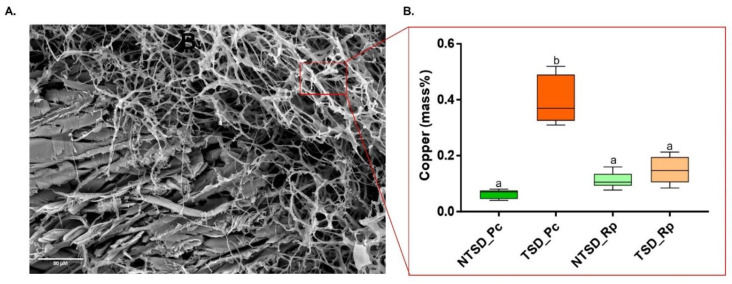
SEM-EDS-based copper quantification in fungal hyphae of *P. chrysosporium* and *R. placenta* after 10 days in the microcosm with TSD or NTSD. (**A**) SEM image of *P. chrysosporium* hyphae. Zones only focusing on mycelium and avoiding detecting copper in the wood substrate were selected for analysis. (**B**) Copper amount in fungal hyphae (mean ± s.e., *n* = 9, one-way ANOVA and Tukey’s post hoc test (*p*-value ≤ 0.05), different letters to show statistically significant differences).

**Figure 5:**
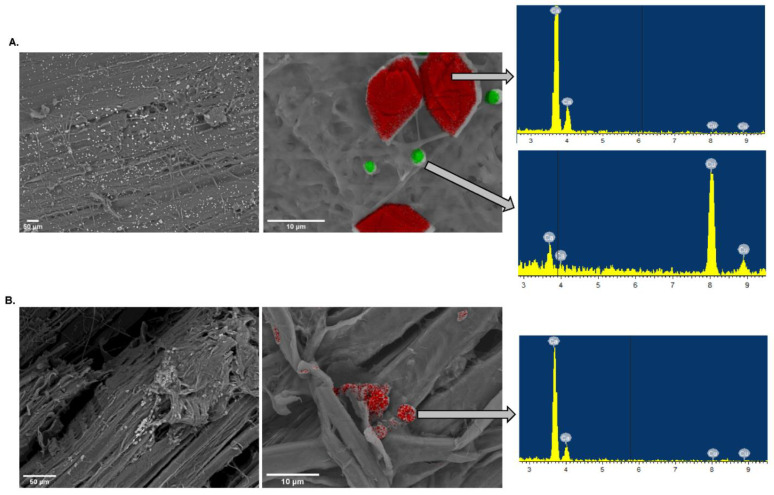
SEM images of oxalate crystals after 10-day culture of *P. chrysosporium* and *R. placenta* with TSD. (**A**) Oxalate crystals produced by *R. placenta*. Red crystals (diamond shape) and green crystals (ball shape) represent calcium and copper oxalate crystals, respectively. (**B**) Oxalate crystals produced by *P. chrysosporium*. Only calcium oxalate crystals were detected.

To better understand how *R. placenta* and *P. chrysosporium* resist the toxicity of the high amounts of Cu within the treated sawdust, Cu was quantified in the hyphae of both fungi. Since the solid phase of the setup is a complex matrix of embedded wood particles and hyphae (Figure S1), SEM coupled with EDS microanalyses were used to quantify the Cu in the hyphae independently from the wood particles (Figure 4A). Cu accumulation was measured in the *P. chrysosporium* (TSD_Pc) hyphae, suggesting the mycelial biosorption of the metal within the cell wall and/or the internalization of Cu within *P. chrysosporium* cells (Figure 4B). Contrary to classical Cu quantification performed on the whole fungal sample, the SEM-EDS approach allowed us to discriminate the Cu biosorbed onto the fungal hyphae from the Cu immobilized at the surface in the form of copper-acid crystals. The Cu levels of *R. placenta* (TSD_Rp) hyphae remained very low; however crystals of two different shapes associated with the *R. placenta* fungal hyphae: ball and diamond shape, were detected (Figure 5A). Microanalyses identified the ball shape (green) as copper oxalate crystals and the diamond shape (red) as calcium oxalate crystals. The same analyses on *P. chrysosporium* showed only a few oxalate crystals. However, no Cu-oxalate crystals were observed, and the few crystals that were detected were Ca-oxalate crystals (Figure 5B). Overall, these results confirm that in the case of Tanalith E-treated wood, *P. chrysosporium* likely uses biosorption at the cell wall as one mechanism to ensure resistance to copper. In contrast, *R. placenta* uses oxalate for extracellular Cu immobilization. However, it is unlikely that the immobilization Cu-oxalate crystals was used as a single mechanism by *R. placenta* for Cu tolerance. Indeed, the strong oxalate-dependent bioleaching released high amounts of soluble Cu in the liquid phase of the microcosm with TSD that the fungus has to cope with.

### 3.4. Comparative Genomics of Copper-Related Genes in R. placenta and P. chrysosporium

**Figure 6:**
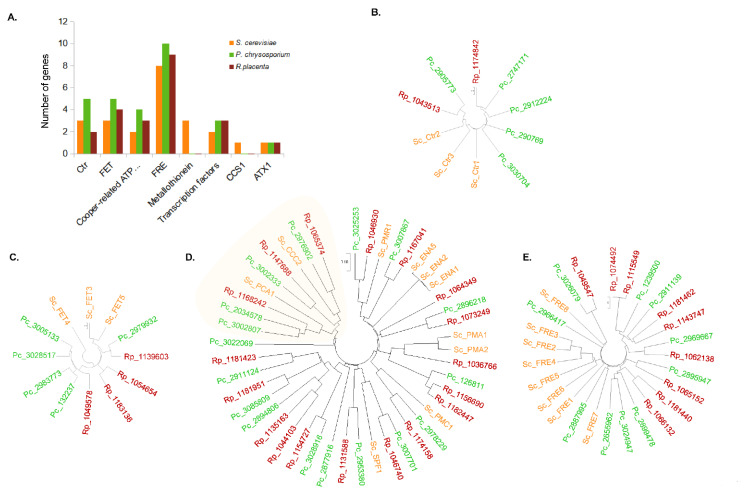
Comparative genomic analysis of copper-related genes in *P. chrysosporium, R. placenta,* and *S. cerevisiae*. (**A**) Number of genes coding for the well-known copper-related proteins in *S. cerevisiae*, related to copper transporters (Ctr, FET, ATPases and FRE), intracellular chelating proteins (Metallothionein), transcription factors, the copper chaperone for superoxide dismutase CCS1, and the cytosolic copper metallochaperone ATX1. Phylogenetic relationship between *S. cerevisiae* (in orange), *R. placenta* (in red), and *P. chrysosporium* (in green) genes coding for Ctr (**B**), FET (**C**), P-ATPases (**D**), and FRE (**E**) orthologues.

To obtain an overview of the putative mechanisms involved in the cell response of *R. placenta* and *P. chrysosporium* to copper, we performed a comparative genomic analysis focused on copper-related genes (Figure 6). Since Cu homeostasis is well-studied in *S. cerevisiae*, a search of orthologues was conducted in the genomes using *S. cerevisiae* sequences as a template. *R. placenta* and *P. chrysosporium* contain a higher number of genes related to Cu transport compared to *S. cerevisiae,* and interestingly, *P. chrysosporium* exhibits more genes coding for the Cu transporters from the CTR, FET, and ATPases families, such as the ferric reductase and cupric reductase from the FRE family compared to *R. placenta* (Figure 6A). ScCtr2 is a low-affinity Cu transporter of the vacuolar membrane [28]. Only one orthologue of ScCtr2 was found in *R. placenta* and *P. chrysosporium*. *P. chrysosporium* exhibits four sequences, (compared to one in *R. placenta*) related to the high-affinity Cu transporters ScCtr1 and ScCtr3 in the plasma membrane (Figure 6B). FET proteins participate in Cu transport. In yeast, ScFET3 and ScFET5 have been characterized as multicopper oxidases that oxidize ferrous iron (Fe^2+^) to ferric iron (Fe^3+^) for subsequent cellular uptake by transmembrane permease Ftr1p [29]. These proteins are required for high-affinity iron uptake and are involved in mediating resistance to Cu ion toxicity. ScFET4 is described as a low-affinity Fe^2+^ transporter of the plasma membrane [30]. Four and five FET-related sequences were detected in the *R. placenta* and *P. chrysosporium* genomes, respectively, compared to three in *S. cerevisiae* (Figure 6C). Ion-transporting P-type ATPases belong to an extended family of transporters. In *S. cerevisiae*, ScCCC2 was identified as being specific to Cu transport and PCA1, which is defined as a cadmium-transporting P-type ATPase, and may also have a role in Cu and iron homeostasis [31]. Figure 6D shows the phylogenetic relationship between the P-ATPase genes. The ones related to Cu transport are underlined in pale yellow. Two orthologues of ScCCC2 were identified in both the *R. placenta and P. chrysosporium* genomes, while one and two orthologues of ScPCA1 were identified in these genomes, respectively (Figure 6D). FRE proteins have been described as ferric reductases and cupric reductases that reduce siderophore-bound iron and oxidized Cu prior to uptake by transporters. In *S. cerevisiae*, FRE1 and FRE2 have been described as being able to oxidize Cu prior to uptake [32]. With few exceptions, sequences from yeast cluster together, and sequences for both basidiomycetes cluster separately, suggesting divergence among this protein family (Figure 6E). Another interesting difference between *S. cerevisiae* and the two basidiomycetes is that no gene coding for metallothionein was detected in the latter genomes. Metallothioneins are small proteins that are rich in cysteines that act in intracellular Cu scavenging. They have been proposed to be one of the two main mechanisms responsible for Cu tolerance in *S. cerevisiae* [19]. This is obviously not the case in basidiomycetes. Globally, this genomic analysis suggests that the difference in gene numbers between *S. cerevisiae* and the two basidiomycetes but also between *R. placenta* and *P. chrysosporium* could reflect the divergence in the cellular response for regulating Cu transport between these fungi.

## 4. Discussion

Fungicides such as Tanalith E used for wood preservation contains a combination of Cu and azoles that inhibit the development of wood-degrading microorganisms by disrupting different basic metabolic processes [5,8,33]. Yet, as illustrated by our study and previous ones [21,34,35], some wood-degrading fungi are able to cope with these toxic compounds. In the present case, SEM microscopic images and respiration measurements indicated the successful colonization of the treated sawdust in the microcosm by both the fungi without impairing fungal growth and primary metabolism when using the preservative treatment. However, while both fungi were able to grow well in the presence of Tanalith E, the resistance mechanisms to Cu toxicity deployed by the two fungi differed drastically. Our data suggest that in the presence of Tanalith E, the resistance of the brown-rot fungus *R. placenta mainly* relied on the immobilization of Cu by oxalic acid, while the white-rot fungus *P. chrysosporium* used biosorption to resist Cu toxicity. Such behaviors are consistent with previous studies since biosorption and metal precipitation are the two main mechanisms used by fungi to tolerate or survive against different toxic metal stresses [11,22,36,37,38,39].

White- and brown-rot fungi differ in their ability to degrade wood. White-rot fungi are unique in terms of lignin degradation, while brown-rot fungi circumvent lignin to degrade holocellulose via iron-dependent oxidative chemistry [12,40,41]. Both groups of fungi can produce oxalate during wood degradation, although at variable levels depending on the species, and oxalic acid secretion may promote wood decay by reducing pH and mobilizing iron [36,42]. In addition, some fungi, especially brown rots, have been shown to be Cu tolerant due to high oxalate production [43,44]. Oxalic acid produced by fungi accumulates as oxalate salt crystals on the outside of fungal hyphae and work as metal chelators rendering the Cu ion inert [40,45]. Likewise, SEM coupled to EDS microanalyses on the Cu-oxalate crystals associated with the fungal hyphae of *R. placenta* identified higher levels of Cu incorporated inside the Cu-oxalate crystals and very low levels of Cu present on the wood. Similar observations were made with *Fomitopsis palustris*, for which microscopic observations of the mycelial mat scraped off from wood blocks highlighted Cu-oxalate complexes at the interface between both fungal mats and the wood surfaces [46]. If oxalic acid was constitutively produced by the fungus in malt extract in our experimental conditions, the levels of oxalate produced by *R. placenta* were two times higher in the presence of TSD compared to NTSD (data not shown), suggesting that the oxalate production by *R. placenta* not only occurred during primary metabolic process but was also stimulated in the presence of copper, likely for copper-based fungicide tolerance.

Interestingly, the ICP and XRF results showed high levels of Cu in the liquid phase, indicating that not all the Cu was immobilized in the Cu-oxalate crystals or was alternatively released from the crystals due to physicochemical modifications in the medium (pH or metabolite secretion for example). These results suggest that the oxalate-mediated chelation of Cu by *R. placenta* was able to mobilize Cu from the wood surface to the liquid phase. These results confirm the strong bioleaching of Cu by *R. placenta,* leading to Cu removal from the wood. Overall, the levels of soluble oxalate were directly correlated with the ability to cleanse Cu-treated wood. However, since the oxalate-dependent bioleaching activity of *R. placenta* released Cu in the liquid phase, it is likely that the complexation of Cu in Cu-oxalate crystals was not sufficient to explain the resistance phenotype of the fungus towards copper, since under these conditions, Cu is still present in high amounts in the liquid phase that is in direct contact with the hyphae. Therefore, it is thus unlikely that a single mechanism was employed by the fungus for Cu tolerance when grown on treated wood.

The ability to produce oxalic acid varies according to the fungal species. Likewise, we found very low levels of oxalate in the *P. chrysosporium* microcosm after 10 days of culture on TSD. Multiple studies on white-rot fungi, especially *P. chrysosporium,* have showed their ability to degrade oxalate rather than to produce and secrete this acid [47,48], and this could be dependent on the culture conditions. The factors affecting oxalate production are primarily the carbon and nitrogen sources in the culture medium and the pH of the environment [49]. When nutrients are plentiful, such as in cultures grown under high-carbon and -nitrogen conditions, no oxalate can be detected in cultures of *P. chrysosporium* [50]. Since the microcosm contained 1 % malt, this could be the reason for the low level of oxalate production by *P. chrysosporium* in this condition. However, the amount of oxalic acid produced did not necessarily indicate the degree of Cu tolerance exhibited by the fungi. For example, *R. placenta* produced higher levels of oxalic acid than *Fibroporia (Poria) vaillantii* but was less Cu tolerant, suggesting that other mechanisms were also involved [51]. Accordingly, we demonstrated that *P. chrysosporium* is tolerant to Tanalith E-treated sawdust despite the lack of oxalate production. One of the probable mechanisms of resistance could be the biosorption ability of this species, which was highlighted by SEM coupled to EDS microanalyses. The high capacity for biosorption was already demonstrated for *P. chrysosporium*, with most studies describing the biosorption of metals, and water contaminants in particular, from aqueous systems [22,52,53,54]. There is limited research on the mechanisms and performance for Cu biosorption in fungi. Indeed, biosorption is a complex mechanism influenced by many physico-chemical and biological factors. The fungal cell wall can act as a cation exchanger, and Cu^2+^ ions can bind or be complexed by carboxylic, phosphate, amine, or sulfhydryl groups of proteins at the cell surface [55]. A high fungal biomass is thus correlated with a high capacity for biosorption [56]. However, no relationship between mycelial growth among different fungal species and the removal of the metal elements present in chromated Cu arsenate-treated wood was found [57]. This points out that this process is part of the Cu resistance mechanism but not of the Cu remediation process. This is consistent with our results since we quantified Cu accumulation in *P. chrysosporium* hyphae but no Cu removal from the treated wood. The biosorbed Cu was thus likely the soluble Cu from the solution due to leaching from TSD after autoclave sterilization in the malt medium. The existence of mucilaginous sheaths around hyphae can also provide a matrix for fungus–mineral interactions and metal transformations harbouring mineral-weathering and metal-chelating agents. Accordingly, the extracellular polymeric substances (EPS) of *P. chrysosporium* were shown to contribute to Pb stress resistance by Pb immobilization [58,59]. Metal diffusion and the precipitation of metal oxalates that occur in this well-hydrated mucilaginous microenvironment were successfully observed using the wet-mode environmental SEM (ESEM) technique [60]. However, this structure is not easily observed by conventional SEM in high-vacuum mode.

Cu can also accumulate inside the cells. The transport of the metal across the cell membrane yields intracellular accumulation, which is dependent on the cell metabolism. This means that this kind of intracellular biosorption may only take place with viable cells. It is often associated with an active defence system of the microorganism, which reacts in the presence of toxic metal [61]. Interestingly, the analysis of *R. placenta* and *P. chrysosporium* genomes highlighted additional Cu transporter genes compared to what has been described in *S. cerevisiae*. Metallothioneins (MT) have a crucial role in the intracellular sequestration of copper. In basidiomycetes, such proteins have been functionally characterized in *Hebeloma cylindrosporum* [62], *Cystoderma carcharias* [63] or *Laccaria bicolor* [64], and sequences have been detected in other Basidiomycete species such as *Pisolithus arhizus*, *Paxillus involutus*, or *Agaricus bisporus* [62]. Surprisingly, these sequences, which were identified in mycorrhizal species, were not found in the *R. placenta* and *P. chrysosporium* genomes, suggesting that either such proteins are absent in these wood decay fungi or, alternatively, are too divergent to be detected by our approach. The only MT detected in *R. placenta* and *P. chrysosporium* genomes is a Cu-thionein that is functionally characterized in the ectomycorrhizal fungus *Suillus luteus* [65]. Because this MT is ubiquitous in the subphylum Agaricomycotina and displays highly conserved features, it has been proposed to be important for basic cellular functions. Its role in Cu tolerance has been demonstrated in *S. luteus*; however, nothing is known concerning the *R. placenta* and *P. chrysosporium* isoforms. Further analysis will be necessary to test whether these proteins are involved in Cu resistance in the two fungi and act as a complement to the oxalate and biosorption protection mechanism.

## 5. Conclusions and Perspectives

In our study, the extracellular production of oxalic acid by *R. placenta* could help fungi to precipitate Cu from treated wood waste, reducing its toxicity. This ability to precipitate strong amounts of Cu from treated solid wood in a short time is quite commendable for copper extraction and thus for the bioremediation of the treated wood. In contrast, *P. chrysosporium* used copper biosorption from the liquid phase to protect itself. Despite it not being as efficient as bioleaching, the ability of *P. chrysosporium* to resist and degrade several organic pollutants is of great interest in the context of wood preservation. Indeed, in the current work, we focused on the fungal ability to detoxify or tolerate Cu levels while Tanalith E also contains azoles that also need to be degraded to obtain full bioremediation. The combination of fungi with different abilities may be a powerful tool to valorize copper azole-containing wood wastes. Finally, both the fungi have the capability to produce a large number of enzymes, transporters, and siderophores that could be further studied to understand the role of these pathways in the detoxification process. The identification of new biocatalysts from these fungi could possibly help us to successfully remove the toxic compounds from wood waste.

## Data Availability

Not applicable.

## Supplementary Materials

The following supporting information can be downloaded at https://www.mdpi.com/article/10.3390/jof8070706/s1, Figure S1: Picture of the microcosm containing *P. chrysosporium* and treated sawdust. Supplemental Data S1: Distribution of sites of interest and microanalysis procedure.
