# Supplementary material for: Comparative Copper Resistance Strategies of Rhodonia placenta and Phanerochaete chrysosporium in a Copper/Azole-Treated Wood Microcosm
Source: J Fungi (Basel). 2022 Jul 4;8(7):706. doi: 10.3390/jof8070706 (PMC9320278; doi:10.3390/jof8070706)
Supplement: Supplementary file 1 [file jof-08-00706-s001.zip › jof-1789081-supplementary.pdf]

Figure S1. Picture of the microcosm containing *P. chrysosporium* and treated sawdust.

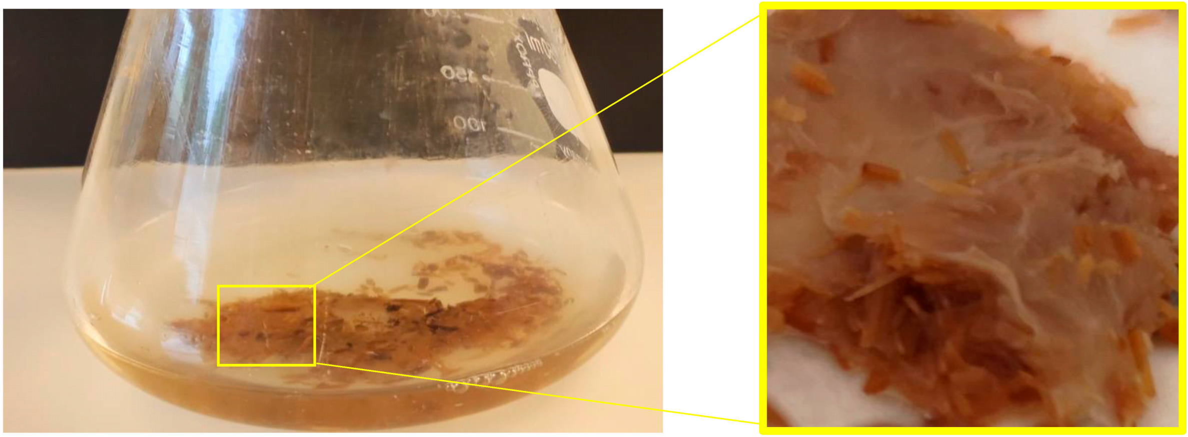

Supplemental Data S1. Distribution of sites of interest and microanalyses procedure.

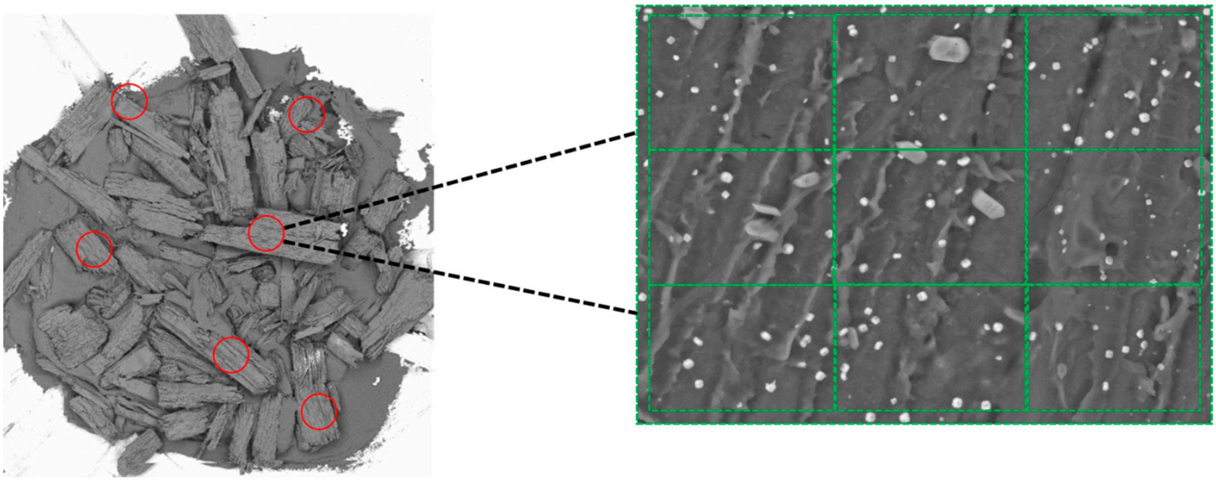

On the left, one sample of treated sawdust incubated with *Rhodonia placenta*. Six different sites of interest, distributed on the stub surface, were highlighted by the red circles. Three sites were chosen to analyze wood, and three others were chosen to analyze the hyphae (not seen at this magnification).

On the right, a higher magnification of one site of interest. Ten spectra were obtained by site of interest, as represented by the green squares.

In total on this stub, 60 spectra were obtained, meaning that 180 spectra were obtained for one condition as there were 3 stubs (samples) by condition.

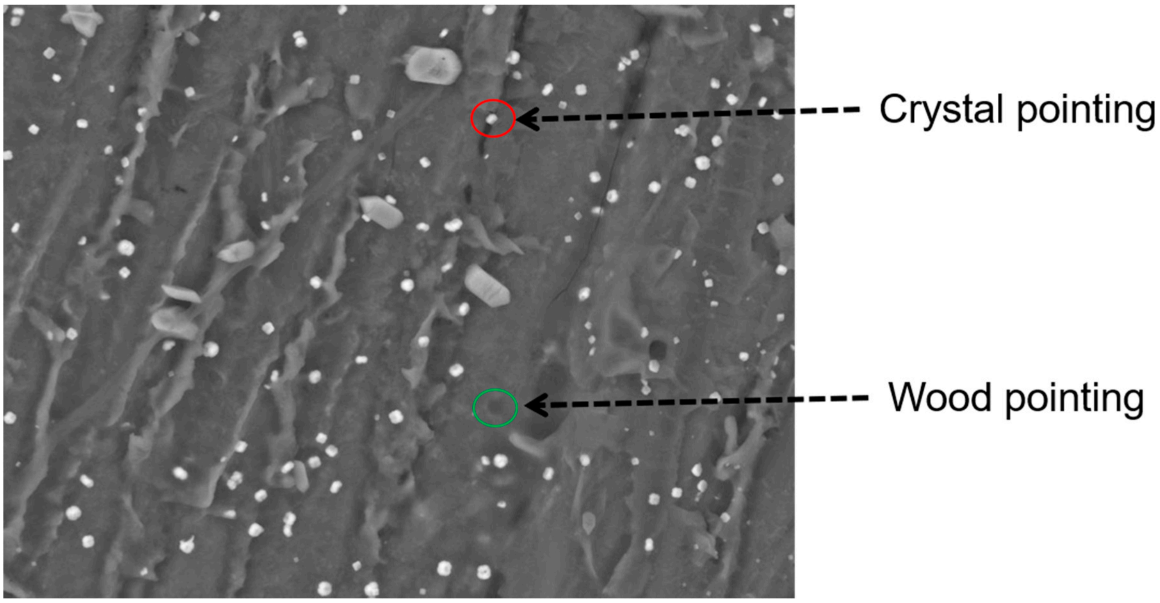

To decipher between copper in wood and copper in crystals, pointing analyses were performed at a higher magnification. 5 points on wood and 5 points on crystals were obtained by site of interest, elevating the number of spectra obtained in total for each sample.
